# Precision Medicine for Relapsed Multiple Myeloma on the Basis of an Integrative Multiomics Approach

**DOI:** 10.1200/PO.18.00019

**Published:** 2018-08-08

**Authors:** Alessandro Laganà, Itai Beno, David Melnekoff, Violetta Leshchenko, Deepu Madduri, Dennis Ramdas, Larysa Sanchez, Scot Niglio, Deepak Perumal, Brian A. Kidd, Riccardo Miotto, Jane Houldsworth, Rita Shaknovich, Ajai Chari, Hearn Jay Cho, Bart Barlogie, Sundar Jagannath, Joel T. Dudley, Samir Parekh

**Affiliations:** **Alessandro Laganà**, **Itai Beno**, **David Melnekoff**, **Violetta Leshchenko**, **Deepu Madduri**, **Dennis Ramdas**, **Larysa Sanchez**, **Scot Niglio**, **Deepak Perumal**, **Brian A. Kidd**, **Riccardo Miotto**, **Ajai Chari**, **Hearn Jay Cho**, **Bart Barlogie**, **Sundar Jagannath**, **Joel T. Dudley**, and **Samir Parekh**, Icahn School of Medicine at Mount Sinai, New York, NY; and **Jane Houldsworth** and **Rita Shaknovich**, Cancer Genetics, Rutherford, NJ.

## Abstract

**Purpose:**

Multiple myeloma (MM) is a malignancy of plasma cells, with a median survival of 6 years. Despite recent therapeutic advancements, relapse remains mostly inevitable, and the disease is fatal in the majority of patients. A major challenge in the treatment of patients with relapsed MM is the timely identification of treatment options in a personalized manner. Current approaches in precision oncology aim at matching specific DNA mutations to drugs, but incorporation of genome-wide RNA profiles has not yet been clinically assessed.

**Methods:**

We have developed a novel computational platform for precision medicine of relapsed and/or refractory MM on the basis of DNA and RNA sequencing. Our approach expands on the traditional DNA-based approaches by integrating somatic mutations and copy number alterations with RNA-based drug repurposing and pathway analysis. We tested our approach in a pilot precision medicine clinical trial with 64 patients with relapsed and/or refractory MM.

**Results:**

We generated treatment recommendations in 63 of 64 patients. Twenty-six patients had treatment implemented, and 21 were assessable. Of these, 11 received a drug that was based on RNA findings, eight received a drug that was based on DNA, and two received a drug that was based on both RNA and DNA. Sixteen of the 21 evaluable patients had a clinical response (ie, reduction of disease marker ≥ 25%), giving a clinical benefit rate of 76% and an overall response rate of 66%, with five patients having ongoing responses at the end of the trial. The median duration of response was 131 days.

**Conclusion:**

Our results show that a comprehensive sequencing approach can identify viable options in patients with relapsed and/or refractory myeloma, and they represent proof of principle of how RNA sequencing can contribute beyond DNA mutation analysis to the development of a reliable drug recommendation tool.

## INTRODUCTION

Multiple myeloma (MM) is a mostly incurable malignancy of terminally differentiated plasma cells that affects 6.5 per 100,000 people per year in the United States, making it the second most common hematologic malignancy.^1^ Typically, the trajectory of MM is characterized by a pattern of recurrent remissions and relapses, with patients becoming increasingly refractory to treatment. Hallmarks of MM include chromosomal translocations and copy number alterations (CNA).^2^ However, the causal drivers of MM pathogenesis are still unclear, and treatment is administered empirically on the basis of recurrence risk rather than genetic events. High-throughput DNA sequencing of patients with MM has revealed wide and remarkable heterogeneity of the mutational spectrum across patients and a complex subclonal structure,^3,4^ suggesting that the use of a personalized therapeutic approach is likely to improve the outcomes for myeloma.^5^

In the past 4 years, we have focused on the design and development of a computational platform for personalized therapy of relapsed and/or refractory MM, on the basis of a comprehensive patient profile generated from DNA and RNA sequencing. Many institutions have now started precision medicine programs aimed at identifying viable therapeutic options for patients with cancer on the basis of specific targetable mutations. This is leading to a landmark paradigm shift in cancer therapy, in which treatment may be administered on the basis of the specific genomic alterations observed in a patient’s tumor, rather than on the tumor histology or tissue type.

Our approach to precision medicine of relapsed MM critically incorporates RNA sequencing–based drug repurposing. In this article, we present our platform and the results obtained from a precision medicine clinical trial with 64 patients with relapsed MM seen at Mount Sinai. We show that our comprehensive approach can benefit patients beyond an approach that is based on mutations alone. Importantly, although our pipeline is designed and tailored for the specific needs of MM therapy, it can provide a general framework for incorporating RNA sequencing–based drug repurposing in oncology.

## METHODS

### Protocol Approvals and Patient Enrollment

The patients were physician referred. Enrollment criteria, which included relapsed myeloma, lack of Food and Drug Administration (FDA)–approved therapeutic options, and a prognosis of 6 months of survival, were approved by the Mount Sinai institutional review board (IRB). Informed written consent was obtained from each patient. As part of the IRB-approved genomics protocol, genomics findings were returned to the patient and treating physician in a standardized report to provide interpretative assistance. Patient enrollment started in February 2014 and ended in February 2016. The study was concluded in September 2017.

### Sample Processing and Sequencing

Bone marrow (BM) aspirates and peripheral blood (PB) were obtained from patients with MM in the study. Tumor genomic DNA and RNA were obtained from CD138^+^ cells isolated from BM (Data Supplement). Normal genomic DNA (control) was obtained from granulocytes isolated from PB. Whole-exome sequencing (WES) and RNA sequencing libraries were submitted to Illumina HiSeq2500 for paired-end sequencing (100 base pairs). Targeted sequencing was performed using the Lymphoma Extended targeted next-generation sequencing panel from Cancer Genetic, Rutherford, NJ; Data Supplement). Raw sequencing data are available at National Center for Biotechnology Information Sequence Read Archive (accession number: PRJNA474747).

### Data Processing and Bioinformatics Analysis

We designed and implemented a software framework for the definition and execution of data analysis workflows. The DNA workflow processes raw data from paired tumor and normal samples to detect, annotate, and prioritize somatic mutations and CNA and to identify actionable alterations. The RNA workflow processes RNA sequencing (RNAseq) data from tumor samples to identify outlier genes, determine pathway activation, and perform drug repurposing. Extended methods are provided in the Data Supplement.

## RESULTS

### Overview of the Study and Patient Characteristics

**Fig 1.**
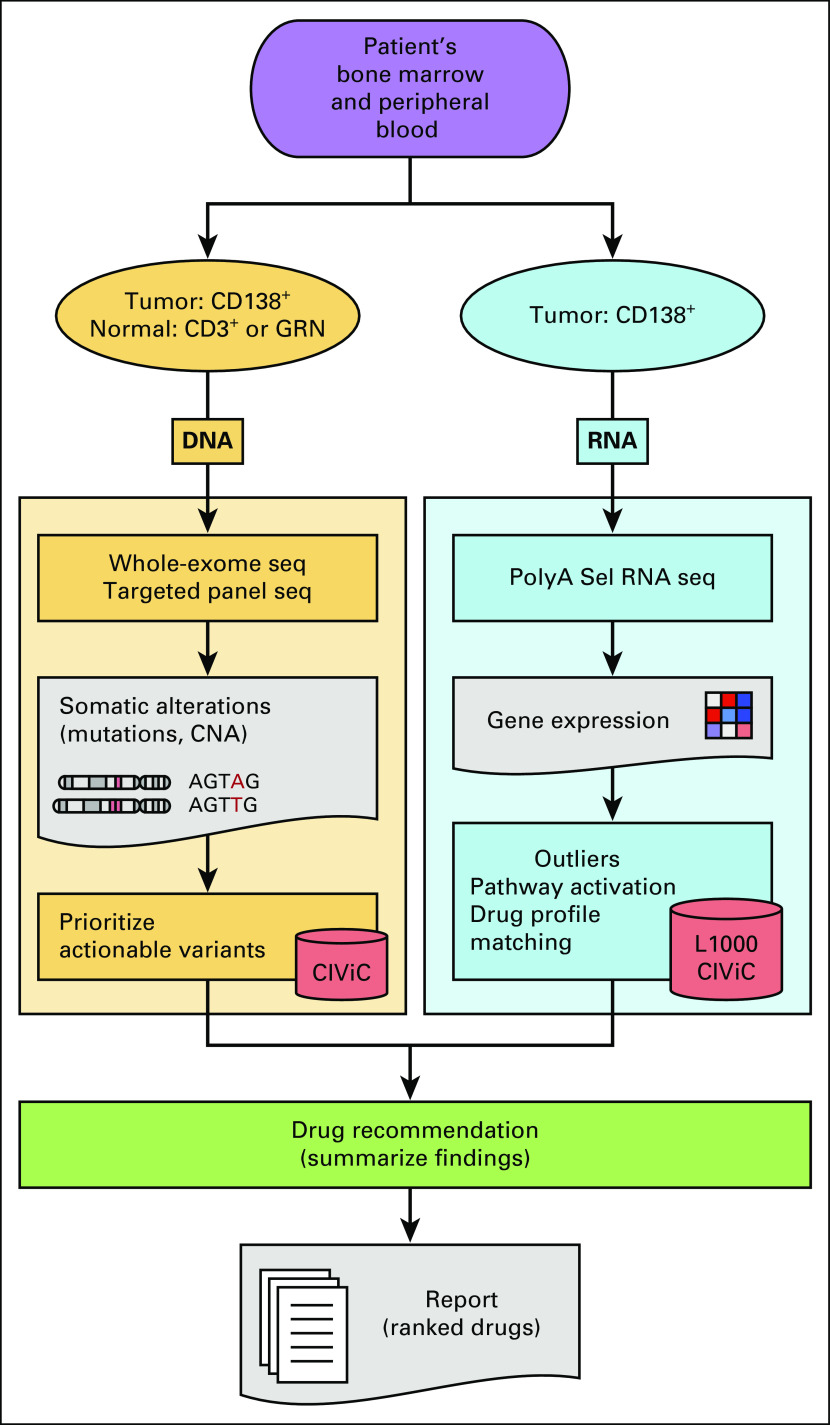
Schema of the analysis pipeline. Left panel (orange) illustrates the DNA processing flow. DNA is extracted from CD138^+^ tumor cells from bone marrow and CD3^+^ or granulocytes (GRN) from peripheral blood as a control. Whole-exome and/or targeted panel sequencing (seq) is performed, and the obtained reads are mapped to the reference genome and analyzed for the identification of somatic mutations and copy number alterations, which are then prioritized on the basis of their actionability. Right panel (blue) illustrates the RNA processing flow. RNA is extracted from CD138^+^ tumor cells, and RNA seq is performed. The obtained reads are mapped to the reference genome and summarized at the gene level. Gene expression analysis is then performed to calculate outlier genes, pathway activation, and drug repurposing through inverse match with drug-induced gene expression profiles. DNA- and RNA-based drug recommendations are then summarized in reports. CIViC, Clinical Interpretations of Variants in Cancer (https://civicdb.org).

We developed a computational platform for personalized therapy of relapsed and/or refractory MM, on the basis of a comprehensive patient profile generated from DNA sequencing and RNAseq data (Fig 1). We evaluated the feasibility and effectiveness of our approach in an IRB-approved precision medicine clinical trial with 64 participants treated at Mount Sinai (See Methods and Table 1). The study included 39 men (61%) and 25 women (39%), and the median age was 59 years (range, 40 to 85 years). Forty-three patients (67%) had high-risk cytogenetics features such as t(4;14), t(14;16), del(13q), del(17p), and gain of 1q (Data Supplement). Overall, the patients had received a median of seven lines of therapy, with 13 patients (20%) having received > 10 lines of therapy.

**Table 1.**
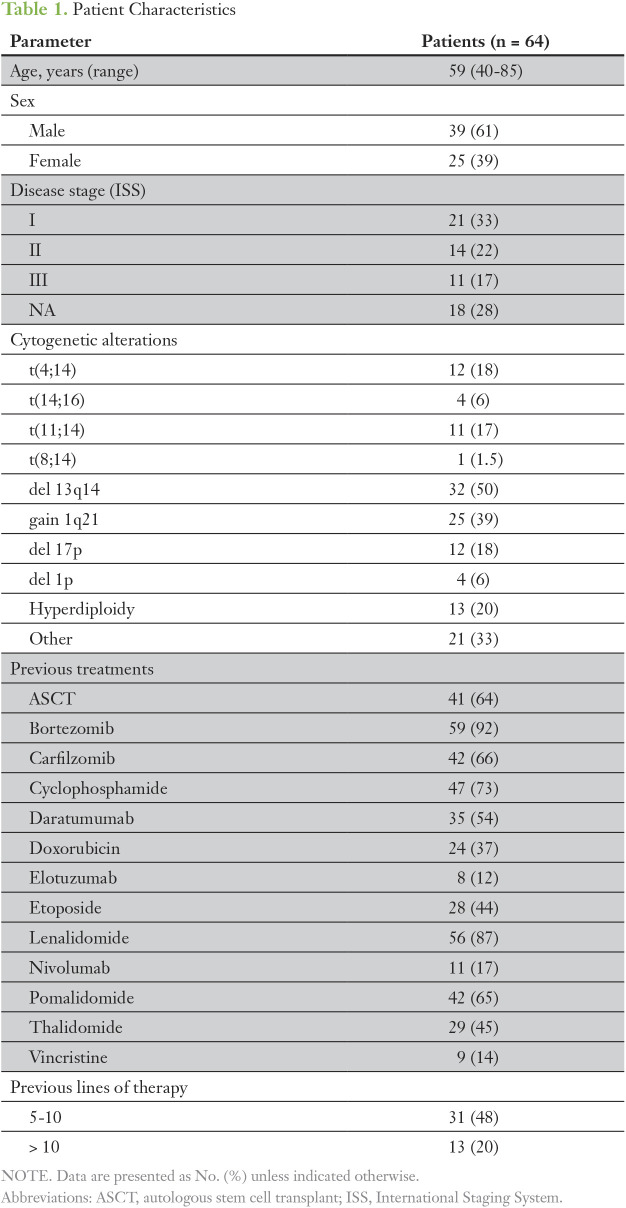
Patient Characteristics

### Genomic Landscape of Patients With Relapsed MM and Actionable Findings

**Fig 2.**
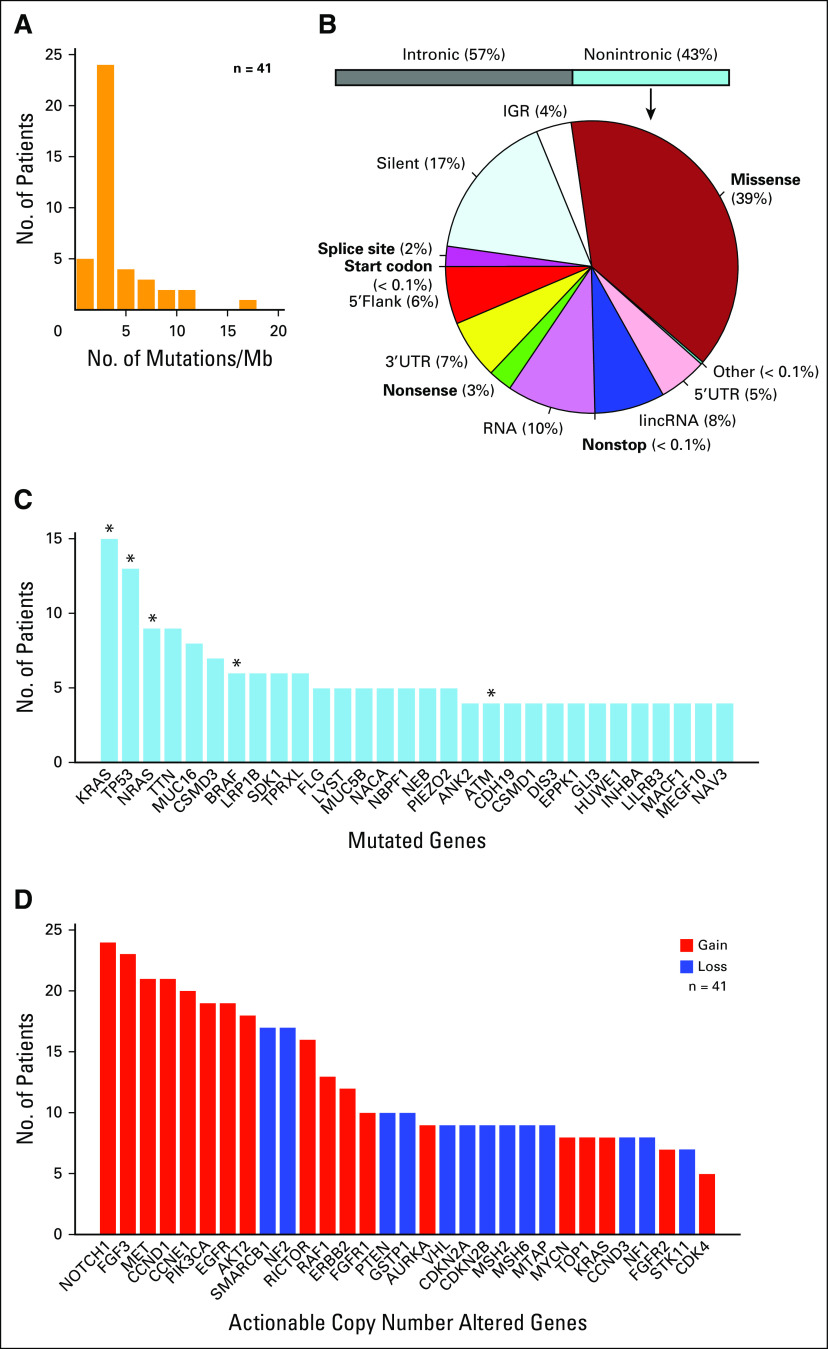
Summary of DNA findings. (A) Distribution of mutational burden (ie, number of total mutations per megabase [Mb] detected; n = 41 patients with whole-exome sequencing [WES] data available). (B) Pie chart illustrates the percentage of nonintronic mutations found per category. Bold print indicates categories of potentially pathogenic mutations. (C) Top 30 mutated genes (n = 55 patients with mutation data available from WES and/or targeted sequencing). (*) Gene carried actionable mutations (according to Clinical Interpretations of Variants in Cancer [https://civicdb.org]). (D) Genes with actionable copy number alterations (according to CIViC) found in the 41 patients with WES data available. The colors indicate that the corresponding gene had mostly gain of copies (red) or loss of copies (blue). IGR, intergenic region; lincRNA, long intergenic noncoding RNA.

We obtained DNA data, either WES or targeted sequencing or both, for 55 patients (86%; Data Supplement). The pipeline identified a total of 21,166 somatic mutations in 10,403 genes in 54 of the 55 patients with DNA data available (Data Supplement). The mutational burden, quantified for the 41 patients with WES data and defined as the total number of mutations, ranged from 113 to 1,423, with an average of 527.5 mutations per patient (Fig 2A). Mutations were distributed among 14 different categories according to their nature and position in the genome, the majority of them being located in introns (57%; Fig 2B). Among the mutations with a potential pathogenic impact, missense mutations were the most numerous (17%), followed by nonsense, splice site, and start codon mutations (< 3%), for a total of 4,013 potentially pathogenic mutations in 3,163 genes (Data Supplement). We used the Clinical Interpretations of Variants in Cancer database (https://civicdb.org) to identify actionable mutations (ie, those associated with sensitivity to one or more drugs in one or more cancer types^6^; Fig 2C; Data Supplement). Among the most frequently mutated genes, KRAS, TP53, NRAS, BRAF, ATM, and APC had actionable mutations. Only the BRAF V600E mutation was associated specifically with MM. The other actionable mutations detected were associated with other hematologic malignancies or with solid cancers.

CNA constitute another important category of abnormalities observed frequently in MM, as well as in other cancers. We estimated CNA for the 41 patients with WES data and summarized them at the gene level. Overall, we found a total of 3,541 genes affected by CNA in at least one patient and identified 31 actionable alterations (Fig 2D; Data Supplement). We assessed concordance of WES-based CNA with fluorescent in situ hybridization and cytogenetics for detection of the most recurrent and clinically relevant CNA in MM (del[1p], del[13q], del[17p], and gain[1q21]) and observed a 69% overlap.

### Analysis of the MM Transcriptome for the Identification of Actionable Variations

**Fig 3.**
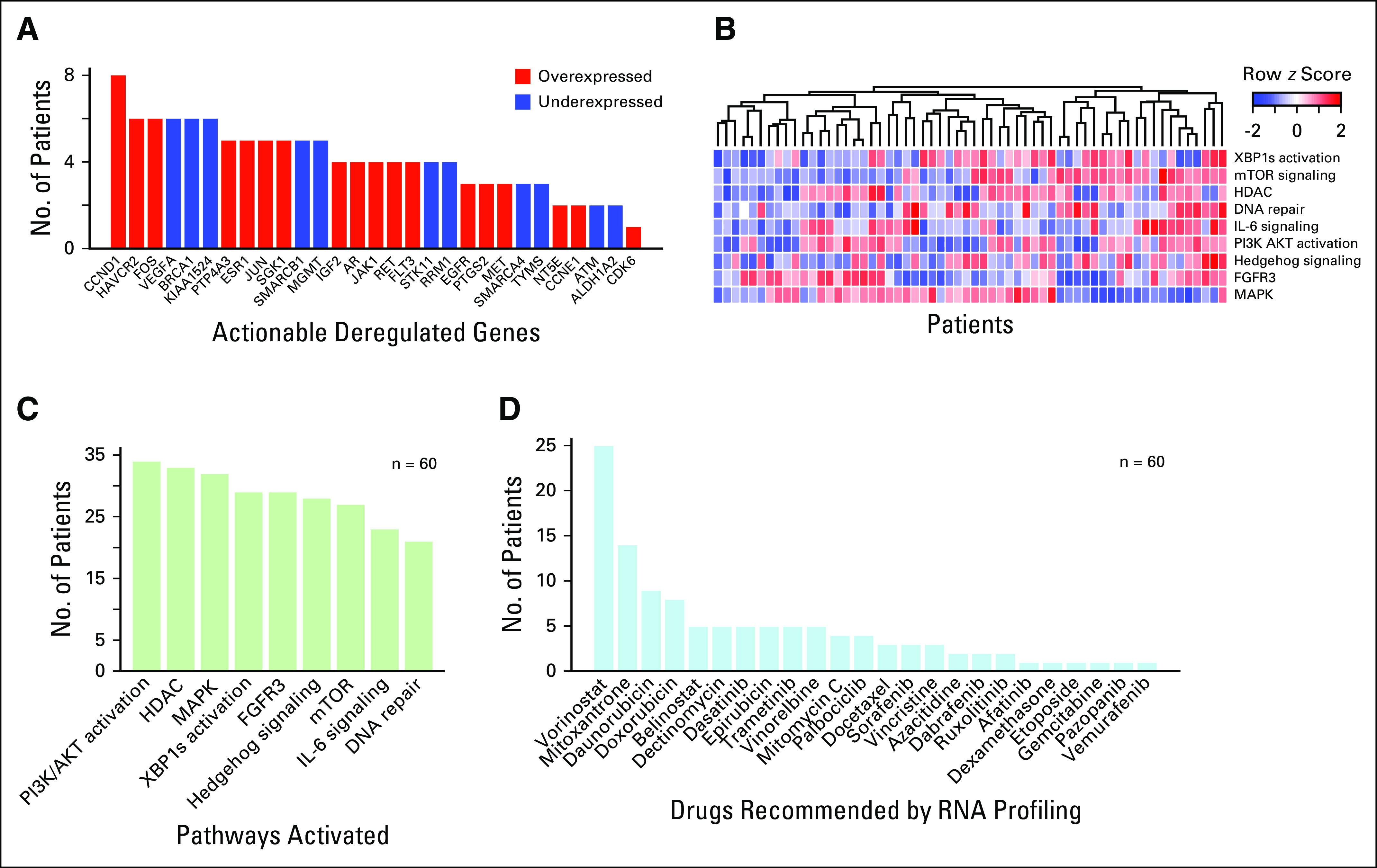
Summary of RNA findings. (A) Actionable outlier genes (according to Clinical Interpretations of Variants in Cancer [https://civicdb.org]). The colors indicate that the corresponding gene was mostly overexpressed (red) or underexpressed (blue). (B) Pathway activation calculated by gene set variation analysis for the 60 patients with RNA sequencing data available. (C) Distribution of activated pathways (ie, positive score) for the 60 patients with RNA sequencing data available. (D) Drugs recommended by RNA-based drug repurposing on the basis of inverse matching of patient gene expression profiles with drug-induced profiles from L1000. FGFR3, fibroblast growth factor receptor 3; HDAC, histone deacetylase; IL-6, interleukin-6; MAPK, mitogen-activated protein kinase; mTOR, mammalian target of rapamycin; P13K AKT, phosphoinositide 3-kinase; XBP1s, Xbox binding protein 1 spliced.

RNAseq data were available for 60 patients (94%) and were used to determine outlier genes and pathway activation and to perform RNA drug repurposing (Data Supplement). We defined deregulated genes (over- or underexpressed) as actionable if they were reported as predictive of sensitivity to at least one drug in CIViC. We identified a total of 28 actionable genes, 17 over- and 11 underexpressed (Fig 3A; Data Supplement). We then calculated pathway activation by single-sample gene set variation analysis^7^ on a set of actionable pathways relevant in MM: XBP1s activation, mammalian target of rapamycin signaling, histone deacetylase (HDAC), DNA repair, interleukin-6 signaling, PI3K/AKT activation, Hedgehog signaling, fibroblast growth factor receptor 3, and mitogen-activated protein kinase (MAPK). Figure 3B presents pathway activation scores calculated for the 60 patients, whereas Figure 3C illustrates the distribution of activated pathways in the cohort. Each pathway is associated with specific targeted drugs (Data Supplement).

Finally, we performed RNA-based drug repurposing by matching each patient’s RNA profile (*z* scores) with gene expression profiles induced by different drugs from the L1000 project, using the L1000CDS2 method, which is based on characteristic direction signatures.^8,9^ The rationale behind this approach is that a drug inducing a gene expression profile that is opposite to a patient’s profile might be able to revert the disease-associated signature and the phenotype. This approach has been demonstrated successfully in several published cases.^10-12^ Figure 3D presents the distribution of FDA-approved cancer drugs selected by our analysis in the cohort. The Data Supplement summarizes FDA-approved noncancer drugs options that were selected for at least one patient.

### Implemented Recommendations and Response to Therapy

Our pipeline generated recommendations for 63 of 64 patients (98%). Recommended drugs were prioritized on the basis of their specific association with MM, according to the available evidence in CIViC (Data Supplement). Of the 63 patients with recommendations, 26 received at least one of the suggested drugs (42%). The most prescribed drugs were trametinib (n = 16 [61%]), venetoclax (n = 8 [30%]), and panobinostat (n = 6 [23%]). Trametinib was recommended because of mutations in either NRAS or KRAS; venetoclax was recommended because of high BCL2 expression in the context of all the patients analyzed (Data Supplement); panobinostat was recommended on the basis of RNAseq analysis indicating activation of the HDAC pathway and/or by RNA-based drug repurposing selecting the pan-HDAC inhibitor vorinostat. Other prescribed drugs were dabrafenib, prescribed because of concurrent BRAF and RAS mutations, and etoposide, selected by RNA-based drug repurposing (Table 2).

**Table 2.**
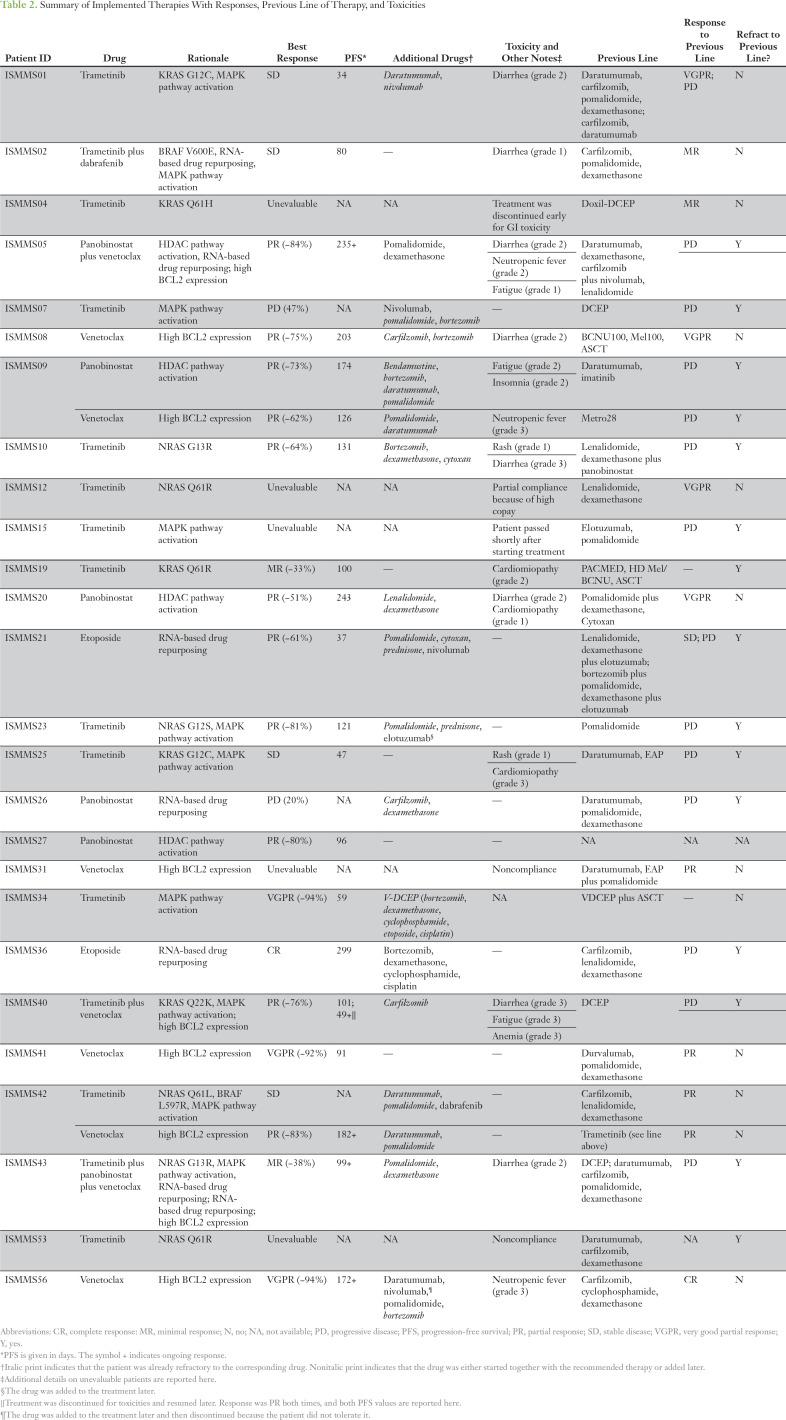
Summary of Implemented Therapies With Responses, Previous Line of Therapy, and Toxicities

**Fig 4.**
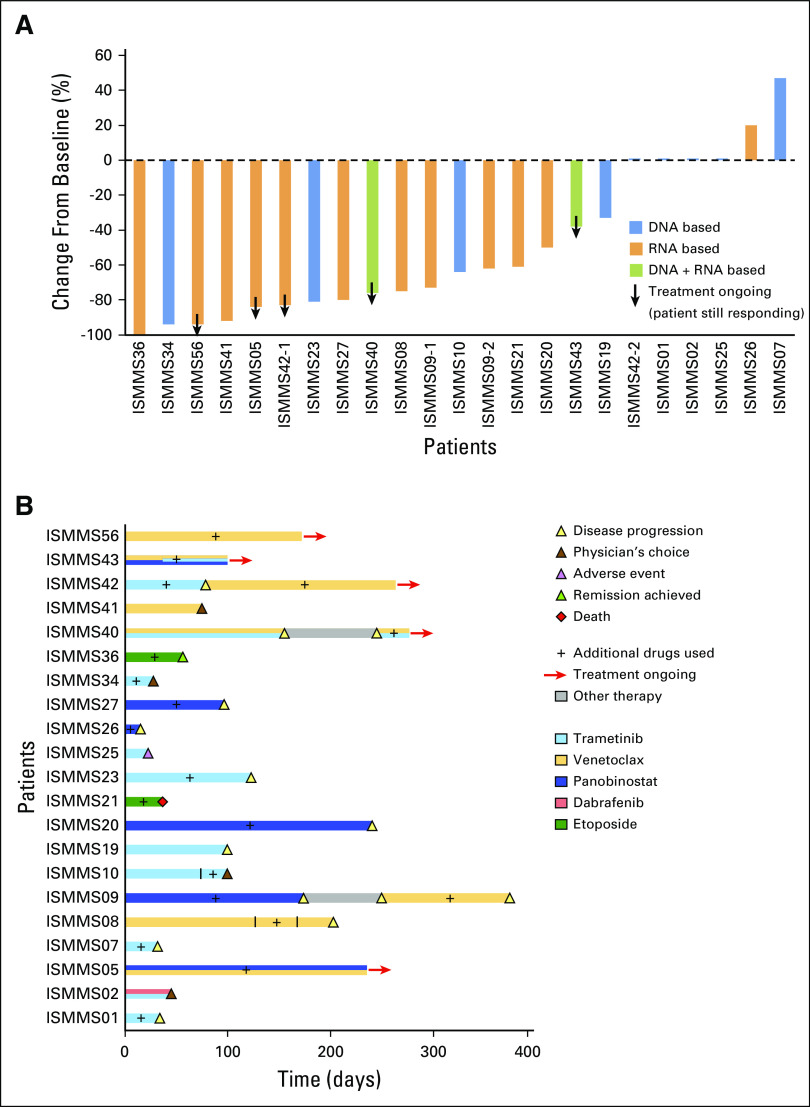
Depth of response and timeline of treatments. (A) Chart shows depth of response as the percentage change from baseline (start of therapy), for patients who received our recommended drugs. Response was determined after the International Myeloma Working Group criteria. Patients ISMMS42 and ISMMS09 received two different drugs at different times; thus, their responses are shown as two separate bars. Colors indicate the source data used to generate the recommendation, either DNA or RNA or both (see legend). The arrow indicates that the patient had an ongoing response at the end of the study. (B) Each bar indicates the time that each patient was receiving our recommended treatment. Each color represents a different drug, coded as follows: trametinib (light blue), venetoclax (dark gold), panobinostat (dark blue), dabrafenib (salmon), and etoposide (dark red). Multicolored portions of the bars indicate time receiving a drug combination on the basis of our recommendation. Triangle indicates discontinuation of treatment, where reasons are color coded as follows: disease progression (gold), physician’s choice (green), adverse event (pink), and remission achieved (white). Red diamond indicates death of the patient. (+) Additional drugs were used in the specific time frame (see Table 2 for details). Gray bars indicate time receiving a different treatment outside of our recommendation. Red arrow indicates patient had an ongoing response at the end of the study.

Of the 26 treated patients, 21 were evaluable for response (81%). Of these, 11 (52%) received a drug on the basis of RNA profiling, eight (38%) on the basis of DNA profiling, and two (10%) on the basis of both RNA and DNA profiling. Five patients received our recommended drugs alone, whereas for 16 patients, the drugs were either added to previous treatment (n = 10) or administered in combination with other drugs on the basis of physician discretion (n = 6). The clinical benefit rate was 76% (minimal response or greater; Fig 4A). In particular, of the 21 evaluable patients, one (5%) achieved CR; three (14%), very good partial response; and 10 (47%), PR, to give an overall response rate of 66%. Two patients (10%) had minimal response, three (14%) had stable disease, and two (10%) had progressive disease. The median duration of response was 131 days (range, 37 to 372 days). Five patients had still ongoing responses at the end of the study (September 1, 2017), with response durations of 235, 182, 150, 172, and 99 days, respectively (Fig 4B). Significant (≥ grade 3) nonhematologic toxicities were seen in five patients (24%); these included neutropenic fever, diarrhea, fatigue, and cardiomyopathy (Table 2).

### Case Studies

#### Case 1: Patient ISMMS05 (RNA-Based Recommendation: Panobinostat Plus Venetoclax).

A 73-year-old woman was diagnosed with IgG lambda MM in November 2007. She received lenalidomide and dexamethasone as front-line treatment, then relapsed and received multiple lines of chemotherapy (Data Supplement). Her CD138^+^ cells were then collected and sequenced. Our pipeline revealed activation of the HDAC pathway through RNA analysis and, concordantly, identified the HDAC inhibitor vorinostat through drug repurposing. Moreover, gene expression analysis revealed a high expression of BCL2 compared with that of the other patients analyzed (Data Supplement). On the basis of these findings, she was administered venetoclax 400 mg PO once daily, the HDAC inhibitor panobinostat 20 mg Monday, Wednesday, and Friday, 2 weeks on, 1 week off, and, in addition, pomalidomide 2 mg Monday to Friday, 3 weeks on, 1 week off. Notably, the patient had been treated previously with pomalidomide. Before therapy, IgG was elevated to 2,910 mg/dL and free lambda, 141. IgG has decreased to as low as 785 mg/dL and free lambda light chains to 19.16 mg/dL (Data Supplement). The patient remains receiving treatment.

#### Case 2: Patient ISMMS23 (WES-Based Recommendation: Trametinib).

A 72-year-old man was diagnosed with IgA kappa plus kappa MM, Durie-Salmon stage IIB in April 2011. After relapsing after receiving multiple treatments including pomalidomide 2 mg (immediate preceding regimen), his CD138^+^ cells and PB samples were sent for sequencing (Data Supplement). The pipeline identified an NRAS G12S mutation, and the patient was administered the MEK inhibitor trametinib. Before treatment, his IgA and free kappa light chains measured 661 mg/dL and 576 mg/L, respectively (free kappa/lambda ratio, 19·32). Three months after treatment began, his IgA had reached a nadir of 94 mg/dL, whereas his free kappa light chains had decreased to 109 mg/L. The patient relapsed 5 months later, with free kappa light chains rising to 390 mg/L (IgA, 187 mg/dL; Data Supplement).

#### Case 3: Patient ISMMS40 (WES + RNA-Based Recommendation: Trametinib Plus Venetoclax).

A 55-year-old man was diagnosed with IgG kappa MM in April 2008. The patient was initially administered lenalidomide and dexamethasone, which resulted in relapse and, after multiple failed regimens, his CD138^+^ cells and PB were sent for sequencing (Data Supplement). WES analysis identified a KRAS Q22K mutation. Concordantly, RNA analysis showed activation of the MAPK pathway. Gene expression analysis revealed a high expression of BCL2 compared with that of the other patients analyzed (Data Supplement). He was administered the BH3-mimetic venetoclax 400 mg Monday to Friday and trametinib 2 mg Monday, Wednesday, Friday. It has been shown that the combination of BH3-mimetic and MEK inhibition upregulates the proapoptotic Bcl-2 family member Bim and can have a synergistic anticancer activity.^13^ The patient’s free kappa/lambda ratio decreased from 13.2 to 0.251, and he responded well to therapy. However, he eventually developed grade 3 fatigue, and treatment was held. After relapse, the patient was challenged with venetoclax 400 mg Monday to Friday, trametinib 2 mg Monday, Wednesday, Friday, and carfilzomib 20/27 mg/m^2^. This showed tumor response, with an M spike decrease from 6.08 g/dL to 4.86 g/dL and an IgG decrease from 7,321 mg/dL to 4,818 mg/dL. Notably, the patient was previously refractory to carfilzomib. The patient has been continuing this regimen for 3 months (Data Supplement).

## DISCUSSION

**Fig 5.**
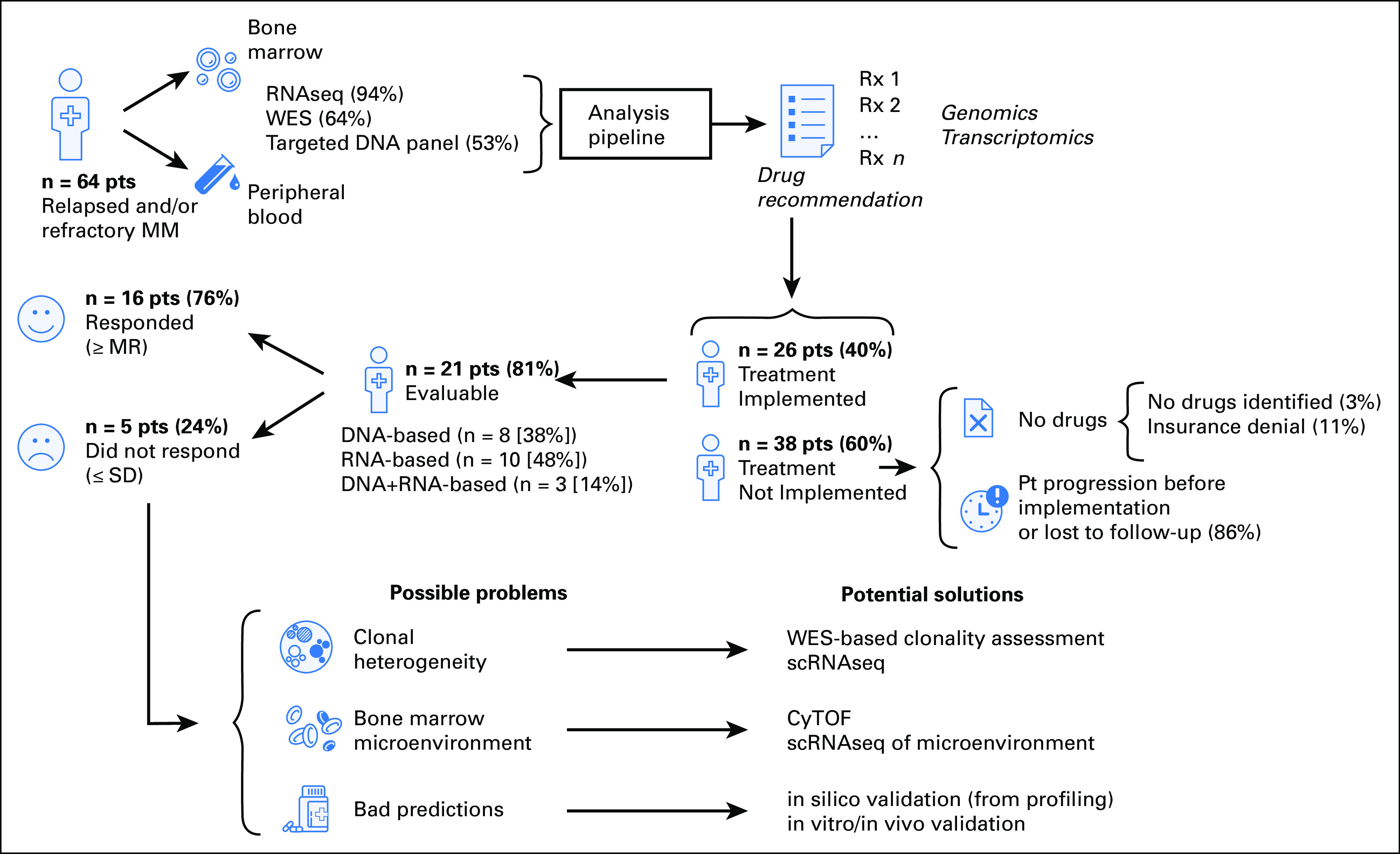
Schematic of the trial with results, limitations, and proposed solutions. We recruited 64 patients (pts) with relapsed and/or refractory multiple myeloma (MM) treated at the Mount Sinai hospital. We obtained RNA sequencing (RNAseq) for 94%, whole-exome sequencing (WES) for 64%, and targeted DNA panel data for 53% of the pts. Sequencing (Seq) data were analyzed by our pipeline (see Fig. 1), and drug recommendation reports were produced. Treatment was implemented in 40% of the pts, and 81% of these were evaluable (see Table 2). According to IMWG criteria, 76% of the pts had a clinical response (MR and above, where MR = minimal response and corresponds to a 25% reduction of disease marker), whereas 24% of the pts had stable disease (SD) or worse. Problems to address to improve recommendations include assessment of clonal heterogeneity, analysis of bone marrow microenvironment, and extension of reference data to include MM-specific drug profiles from in vitro and in vivo models. Treatment was not implemented in 60% of the pts, because either no drugs were identified, because insurance denied the proposed drugs, or because of rapid progression of the patient before the results of sequencing were available. CyTOF, mass cytometry; scRNA, single-cell RNA.

Here, we reported our integrated multiomics approach for personalized therapy of MM and its application in a pilot precision medicine clinical trial with 64 patients with relapsed MM seen at Mount Sinai. As patients with MM progress through advanced disease and receive multiple lines of therapy, they are left with fewer and fewer treatment options. The results of our study show how a precision medicine approach incorporating RNA sequencing may identify viable and effective therapeutic options beyond the current FDA-approved armamentarium in MM. Our pipeline generated recommendations from a larger pool of FDA-approved cancer drugs (MM and non-MM) for 63 of 64 patients in the trial (98%; Fig 5).

Treatment was implemented in 40% of patients, and 81% of these were evaluable (Table 2). Remarkably, 62% of the evaluable patients received a drug on the basis of RNA profiling. Two drugs that we repurposed successfully from other cancers in our study were trametinib and venetoclax, the former selected on the basis of DNA and the latter on the basis of RNA findings. Trametinib is a MEK1/2 inhibitor approved by the FDA in combination with dabrafenib for unresectable or metastatic melanoma and non–small-cell lung cancer carrying mutations in BRAF (V600E/V600K). Recent studies have shown activating mutations in NRAS, KRAS, and BRAF in MM, making the MAPK pathway a significant therapeutic target also in MM.^3-5^ A retrospective study has demonstrated clinical activity of trametinib in patients with MM with RAS-mutated tumors.^14^ Eleven of 21 evaluable patients in our study received trametinib, either alone or in combination with other drugs. Six of them had clinical response, with a median progression-free survival of 110 days, and two patients had ongoing responses at the end of the study. Venetoclax is a BH3 mimetic that acts as a Bcl-2 inhibitor and is approved only for patients with chronic lymphocytic leukemia carrying deletion of 17p.^15^ Phase I clinical trials reported the efficacy of venetoclax in relapsed and/or refractory MM, where patients with high BCL2 expression had a higher ORR than did patients with low BCL2 expression.^16-18^ Our pipeline selected venetoclax for eight patients on the basis of BCL2 expression, and all of them showed clinical response, with a median progression-free survival of 161 days, and five patients had ongoing responses at the end of the study. Overall, these results compare favorably to the efficacy seen in several novel agents for MM, including pomalidomide, daratumumab, and elotumumab, as well as targeted precision medicine approaches such as the NCI-MATCH (National Cancer Institute Molecular Analysis for Therapy Choice) trial, in which patients with specific solid tumors received treatment on the basis of actionable DNA mutations.^19^

Our trial also identified the challenges in implementing NGS-based recommendations in a real-world setting. In 60% of the patients with recommendations, treatment was not implemented, either because of insurance denial of the drug or because of rapid progression of disease before sequencing results were available. Insurance denial represents a significant limitation in the implementation of personalized therapy that is based on genomic findings. Our results suggest a need for specialty pharmacies and insurers to evolve with the technology to optimally help patients. 

The second limiting factor in our study was the time required for sequencing. The average turnaround time from sample collection to sequencing data was 6 weeks. However, most patients with relapsing MM experience rapid progression and need immediate treatment. To address this, we are now using rapid-run sequencing, which provides results in a few days. This may be more expensive initially but may become cost effective with greater use.

Of the 21 treated patients who were evaluable, five did not respond to the recommended therapy. Multiple factors should be considered to improve the accuracy of treatment prediction. One key factor is clonal heterogeneity. MM is characterized by a branching pattern of clonal evolution, in which distinct subclonal tumor populations may evolve by selective pressure of therapy.^5,20,21^ We have recently started to investigate the impact of clonal heterogeneity on therapy selection, by extending our analysis to include clonality assessment on the basis of WES and/or single-cell RNA sequencing (scRNAseq). Integration of both WES and scRNAseq may provide a more comprehensive profile of a patient’s tumor, enabling drug repurposing at the subclonal level.

Another key factor in optimal therapy prediction is the BM microenvironment. MM cells are strongly dependent on the surrounding microenvironment, which promotes their homing, growth, survival, and migration, as well as their resistance to drugs.^22^ Therapies targeting both the cellular and the noncellular BM compartments are available and are currently used in MM, although not in a targeted manner. Profiling the microenvironment by scRNAseq and/or mass cytometry, which allows simultaneous measurements of up to 50 markers at single-cell resolution, may help dissect the nontumor compartment and better inform targeted therapy. We are currently investigating the feasibility of including this feature in the next generation of our platform. In particular, the use of scRNAseq in clinical precision oncology is limited by several challenges, including increased cost compared with bulk sequencing (approximately 10 times higher), high sensitivity to sample purity, and dropouts (ie, undetected transcripts due to low amounts of RNA sequenced within individual cells), as well as data processing and interpretation. In fact, the analysis of scRNAseq data requires the use of specialized tools, which are still in their development stage. Nevertheless, scRNAseq represents a promising and powerful technology that will likely complement current precision medicine strategies in the near future.

A third key factor to consider concerns the accuracy of the predictions. This involves sequencing data, reference data in the knowledge base, and the algorithms used. We are working on extending our knowledge base to incorporate MM-specific data, including transcriptional effects of novel agents, to quickly and effectively translate robust research findings into clinical practice. To further improve drug recommendations, we are implementing validation of the in silico findings using both in vitro (micro-C3) and in vivo (PDX) models.^23,24^ This will also contribute to reducing both the costs and the toxicities associated with ineffective treatments. Finally, we are going to equip our platform with a machine learning flow, which will implement interactive learning techniques to refine the predictions on the basis of therapy outcome and physician’s opinion.

In conclusion, here we have described our sequencing-based precision medicine platform for relapsed and/or refractory MM and reported the results of a pilot clinical trial to assess the feasibility and usefulness of this approach. The trial has allowed us to test and reveal the accuracy of our platform and to understand the pitfalls and limitations of the approach, laying the foundation for our next-generation precision medicine framework. Overall, the results demonstrate feasibility and early efficacy, providing a basis for expanding NGS-guided personalized therapy integrating RNA- and DNA-based drug repurposing for patients with cancer.

## References

[1] Siegel RL Miller KD Jemal A Cancer statistics, 2017 CA Cancer J Clin 67 7 30 2017 28055103 10.3322/caac.21387

[2] Morgan GJ Walker BA Davies FE The genetic architecture of multiple myeloma Nat Rev Cancer 12 335 348 2012 22495321 10.1038/nrc3257

[3] Chapman MA Lawrence MS Keats JJ et al Initial genome sequencing and analysis of multiple myeloma Nature 471 467 472 2011 21430775 10.1038/nature09837 PMC3560292

[4] Lohr JG Stojanov P Carter SL et al Widespread genetic heterogeneity in multiple myeloma: Implications for targeted therapy Cancer Cell 25 91 101 2014 24434212 10.1016/j.ccr.2013.12.015 PMC4241387

[5] Bolli N Avet-Loiseau H Wedge DC et al Heterogeneity of genomic evolution and mutational profiles in multiple myeloma Nat Commun 5 2997 2014 24429703 10.1038/ncomms3997 PMC3905727

[6] Griffith M Spies NC Krysiak K et al CIViC is a community knowledgebase for expert crowdsourcing the clinical interpretation of variants in cancer Nat Genet 49 170 174 2017 28138153 10.1038/ng.3774 PMC5367263

[7] Hänzelmann S Castelo R Guinney J GSVA: Gene set variation analysis for microarray and RNA-seq data BMC Bioinformatics 14 7 2013 23323831 10.1186/1471-2105-14-7 PMC3618321

[8] Campillos M Kuhn M Gavin AC et al Drug target identification using side-effect similarity Science 321 263 266 2008 18621671 10.1126/science.1158140

[9] Duan Q Reid SP Clark NR et al L1000CDS^2^: LINCS L1000 characteristic direction signatures search engine NPJ Syst Biol Appl 2 16015 2016 28413689 10.1038/npjsba.2016.15 PMC5389891

[10] Chen B Ma L Paik H et al Reversal of cancer gene expression correlates with drug efficacy and reveals therapeutic targets Nat Commun 8 16022 2017 28699633 10.1038/ncomms16022 PMC5510182

[11] Dudley JT Deshpande T Butte AJ Exploiting drug-disease relationships for computational drug repositioning Brief Bioinform 12 303 311 2011 21690101 10.1093/bib/bbr013 PMC3137933

[12] Dudley JT Sirota M Shenoy M et al Computational repositioning of the anticonvulsant topiramate for inflammatory bowel disease Sci Transl Med 3 96ra76 2011 10.1126/scitranslmed.3002648 PMC3479650 21849664

[13] Hendrickson AW Meng XW Kaufmann SH Anticancer therapy: Boosting the bang of Bim J Clin Invest 118 3582 3584 2008 18949061 10.1172/JCI37553 PMC2571037

[14] Heuck CJ Jethava Y Khan R et al Inhibiting MEK in MAPK pathway-activated myeloma Leukemia 30 976 980 2016 26228812 10.1038/leu.2015.208 PMC4832073

[15] Roberts AW Davids MS Pagel JM et al Targeting BCL2 with venetoclax in relapsed chronic lymphocytic leukemia N Engl J Med 374 311 322 2016 26639348 10.1056/NEJMoa1513257 PMC7107002

[16] Touzeau C Dousset C Le Gouill S et al The Bcl-2 specific BH3 mimetic ABT-199: A promising targeted therapy for t(11;14) multiple myeloma Leukemia 28 210 212 2014 23860449 10.1038/leu.2013.216 PMC3887407

[17] Kumar S Kaufman JL Gasparetto C et al Efficacy of venetoclax as targeted therapy for relapsed/refractory t(11;14) multiple myeloma Blood 130 2401 2409 2017 29018077 10.1182/blood-2017-06-788786

[18] Moreau P Chanan-Khan A Roberts AW et al Promising efficacy and acceptable safety of venetoclax plus bortezomib and dexamethasone in relapsed/refractory MM Blood 130 2392 2400 2017 28847998 10.1182/blood-2017-06-788323

[19] Brower V NCI-MATCH pairs tumor mutations with matching drugs Nat Biotechnol 33 790 791 2015 26252121 10.1038/nbt0815-790

[20] Bianchi G Ghobrial IM Biological and clinical implications of clonal heterogeneity and clonal evolution in multiple myeloma Curr Cancer Ther Rev 10 70 79 2014 25705146 10.2174/157339471002141124121404 PMC4334389

[21] Brioli A Melchor L Cavo M et al The impact of intra-clonal heterogeneity on the treatment of multiple myeloma Br J Haematol 165 441 454 2014 24580032 10.1111/bjh.12805

[22] Andrews SW Kabrah S May JE et al Multiple myeloma: The bone marrow microenvironment and its relation to treatment Br J Biomed Sci 70 110 120 2013 24273897 10.1080/09674845.2013.11669945

[23] Pak C Callander NS Young EW et al MicroC(3): An ex vivo microfluidic cis-coculture assay to test chemosensitivity and resistance of patient multiple myeloma cells Integr Biol 7 643 654 2015 10.1039/c5ib00071h PMC4476551 25998180

[24] Pauli C Hopkins BD Prandi D et al Personalized *in vitro* and *in vivo* cancer models to guide precision medicine Cancer Discov 7 462 477 2017 28331002 10.1158/2159-8290.CD-16-1154 PMC5413423

